# The “rights” of precision drug development for Alzheimer’s disease

**DOI:** 10.1186/s13195-019-0529-5

**Published:** 2019-08-31

**Authors:** Jeffrey Cummings, Howard H. Feldman, Philip Scheltens

**Affiliations:** 1 Department of Brain Health, School of Integrated Health Sciences, UNLV and Cleveland Clinic Lou Ruvo Center for Brain Health, 888 West Bonneville Ave, Las Vegas, NV 89106 USA; 2 Department of Neurosciences, Alzheimer’s Disease Cooperative Study, University of California San Diego, San Diego, CA USA; 3 Alzheimer Center Amsterdam, Amsterdam University Medical Centers, Amsterdam, The Netherlands

**Keywords:** Alzheimer’s disease, Drug development, Clinical trials, Biomarkers

## Abstract

There is a high rate of failure in Alzheimer’s disease (AD) drug development with 99% of trials showing no drug-placebo difference. This low rate of success delays new treatments for patients and discourages investment in AD drug development. Studies across drug development programs in multiple disorders have identified important strategies for decreasing the risk and increasing the likelihood of success in drug development programs. These experiences provide guidance for the optimization of AD drug development. The “rights” of AD drug development include the right target, right drug, right biomarker, right participant, and right trial. The right target identifies the appropriate biologic process for an AD therapeutic intervention. The right drug must have well-understood pharmacokinetic and pharmacodynamic features, ability to penetrate the blood-brain barrier, efficacy demonstrated in animals, maximum tolerated dose established in phase I, and acceptable toxicity. The right biomarkers include participant selection biomarkers, target engagement biomarkers, biomarkers supportive of disease modification, and biomarkers for side effect monitoring. The right participant hinges on the identification of the phase of AD (preclinical, prodromal, dementia). Severity of disease and drug mechanism both have a role in defining the right participant. The right trial is a well-conducted trial with appropriate clinical and biomarker outcomes collected over an appropriate period of time, powered to detect a clinically meaningful drug-placebo difference, and anticipating variability introduced by globalization. We lack understanding of some critical aspects of disease biology and drug action that may affect the success of development programs even when the “rights” are adhered to. Attention to disciplined drug development will increase the likelihood of success, decrease the risks associated with AD drug development, enhance the ability to attract investment, and make it more likely that new therapies will become available to those with or vulnerable to the emergence of AD.

## Introduction

Alzheimer’s disease (AD) is rapidly increasing in frequency as the world’s population ages. In the USA, there are currently an estimated 5.3 million individuals with AD dementia, and this number is expected to increase to more than 13 million by 2050 [1, 2]. Approximately 15% of the US population over age 60 has prodromal AD and nearly 40% has preclinical AD [3]. Similar trends are seen globally with an anticipated worldwide population of AD dementia patients exceeding 100 million by 2050 unless means of delaying, preventing, or treating AD are found [4]. The financial burden of AD in the USA will increase from its current $259 billion US dollars (USD) annually to more than $1 trillion USD by 2050 [5]. The cost of AD to the US economy currently exceeds that of cancer or cardiovascular disease [6].

Amplifying the demographic challenge of the rising numbers of AD victims is the low rate of success of the development of AD therapies. Across all types of AD therapies, the failure rate is more than 99%, and for disease-modifying therapies (DMTs), the failure rate is 100% [7, 8]. These numbers demand a re-examination of the drug development process. Success in other fields such as cancer therapeutics can be helpful in guiding better drug discovery and development practices of AD treatments. For example, 12 of 42 (28%) drugs approved by the US Food and Drug Administration (FDA) in 2017 were oncology therapies (www.fda.gov); this contrasts with 0% of AD drugs in development. There are currently 112 new molecular entities in clinical trials in AD, whereas there are 3558 in cancer trials [9, 10]. Success in cancer drug development attracts funding and leads to more clinical trials, accelerating the emergence of new therapies. This model can assist in improving AD drug development.

Patient care increasingly demands precision medicine with the right drug, in the right dose, administered to the right patient, at the right time [11–13]. Precision medicine requires precision drug development. Effective medications, delivered in a correct dose, to a patient in the stage of the illness that can be impacted by therapy requires that these precision treatment characteristics be determined in a disciplined drug development program [14]. Drug development sponsors have developed systematic approaches to drug testing including the “rights” of drug development [15, 16], the “pillars” of drug development [17], model-based drug development [18, 19], and a translational medicine guide [20]. These approaches are appropriate across therapeutic areas, and none have been applied specifically to AD drug development. Building on these foundations, we describe a set of “rights” for AD drug development which are aligned with precision drug development. We consider lessons derived from drug development across several fields as well as learnings from recent negative AD treatment trials [14, 17, 21, 22]; we note the areas where success in the “right” principles is pursued. These “rights” for drug development are not all new innovations, but recent reviews of the AD drug pipeline show that they are often not implemented [16, 23, 24]. We consider how the “rights” will strengthen the AD drug discovery and development process, increase the likelihood of success, de-risk investment in AD therapeutic research, and spur interest in meeting the treatment challenges posed by the coming tsunami of patients.

**Fig. 1:**
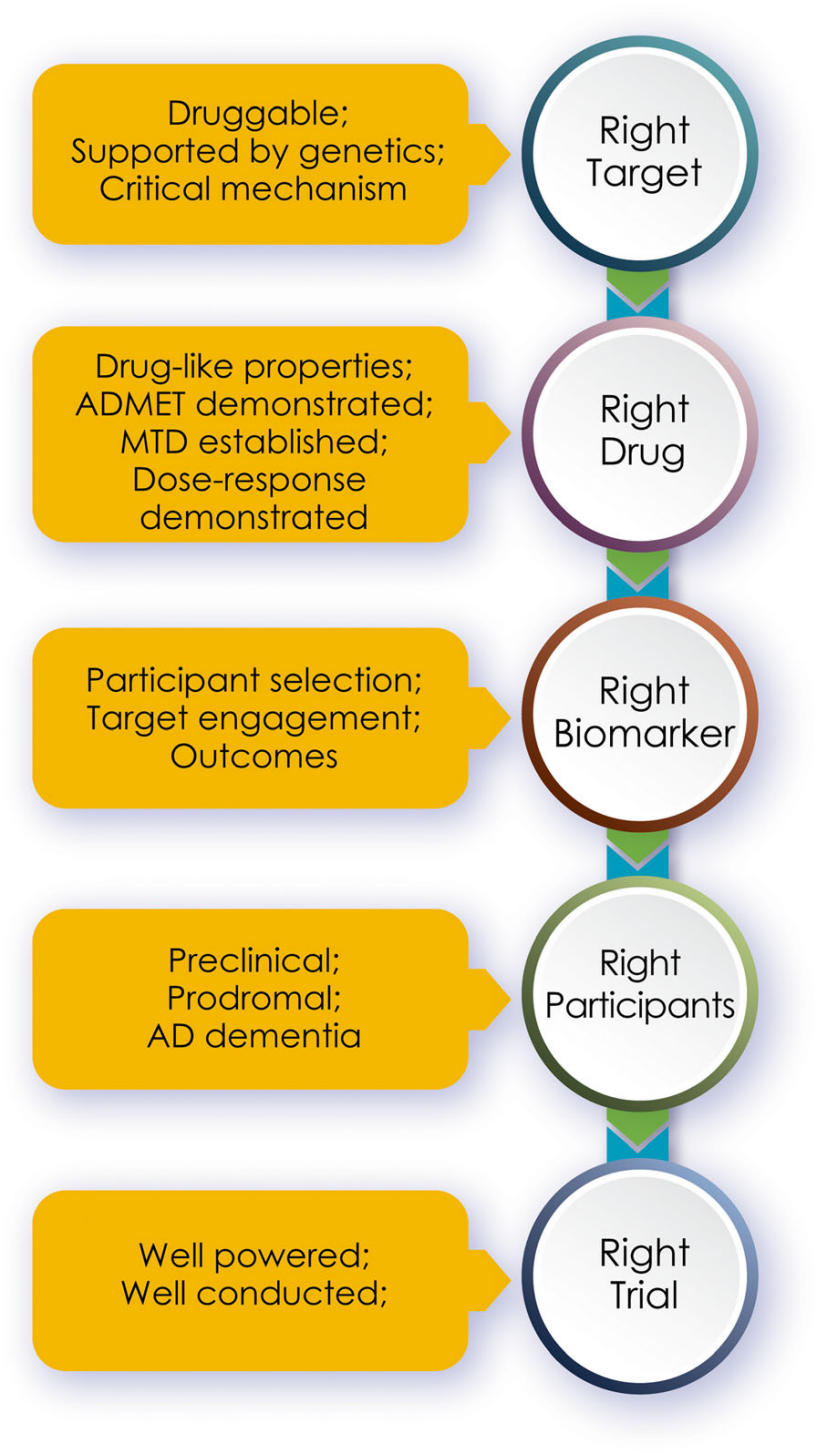
The rights of AD drug development

Figure 1 provides an overview of the “rights of AD drug development.”

## The right target

AD biology is complex, and only one target—the cholinergic system—has been fully validated through multiple successful therapies. Four cholinesterase inhibitors have been found to improve the dual outcomes of cognition plus function or cognition plus  global status in patients with AD dementia [25, 26]. The successful development of memantine supports the validity of the *N*-methyl-d-aspartate (NMDA) receptor as a viable target, although only one agent has been shown to exert a therapeutic effect when modulating this receptor [27, 28]. A combination agent (Namzaric)  addressing these two targets has been approved, establishing a precedent for combination therapy of two approved agents in AD [29]. Cholinesterase inhibitors have shown benefit in mild, moderate, and severe AD dementia [26]; memantine is effective in moderate and severe AD dementia [30]. No agent has shown benefit in prodromal AD (pAD), mild cognitive impairment (MCI), or preclinical AD [31].

No other target has been validated  by successful therapy; all agents currently in development are unvalidated at the level of human benefit. Several targets are partially supported by biological and behavioral effects in animal models, and some agents have shown beneficial effects in preliminary clinical trials [32]. The lack of validation of a target by a specific trial does not disprove its worthiness for drug development; validation depends on concurrent conduct of other “rights” in the development program.

For an agent to be a DMT, the candidate drug treatment must meaningfully intervene in disease processes leading to nerve cell death [33] and be druggable (e.g., modifiable by a small molecule agent or immunotherapy [34, 35]). Viable targets must represent critical non-redundant pathways necessary for neuronal survival. Ideal targets have a proven function in disease pathophysiology, are genetically linked to the disease, have greater representation in disease than in normal function, can be assayed using high-throughput screening, are not uniformly distributed throughout the body, have an associated biomarker, and have a favorable side effect prediction profile [36]. Druggability relates to proteins, peptides, or nucleic acids with an activity that can be modified by a treatment [35].

A current National Institute of Health (NIH) ontology of candidate targets in AD includes amyloid-related mechanisms, tau pathways, apolipoprotein E e-4 (ApoE-4), lipid metabolism, neuroinflammation, autophagy/proteasome/unfolded protein response, hormones/growth factors, dysregulation of calcium homeostasis, heavy metals, mitochondrial cascade/mitochondrial uncoupling/antioxidants, disease risk genes and related pathways, epigenetics, and glucose metabolism [37, 38]. Other mechanisms may emerge; highly influential nodes in networks may be identified through systems pharmacology approaches; and opportunities or requirements for combination therapies may be discovered. Genetic editing techniques are increasingly used in experimental treatment paradigms, and RNA interference approaches show promise in non-AD neurodegenerative disorders [39]. With the recognition that late-life sporadic AD frequently has multiple contributing pathologies, identifying a single molecular therapeutic target whose manipulation is efficacious in all affected individuals may not be forthcoming [40–43].

Analysis of predictors of success in drug development programs shows that agents linked to genetically defined targets have a greater chance of being advanced from one phase to the next than drugs that address targets having no genetic links to the underlying disease [15, 21]. Transgenic (tg) animal models and knockout and knockin models of disease can add to the genetic evidence for a target. Genes can help prioritize drug candidates as well as support target validation [44]. Genes implicate potentially druggable pathways and networks involved in AD pathogenesis [45, 46]. Genetic linkages to amyloid precursor protein (APP), beta-site amyloid precursor protein cleavage enzyme (BACE), gamma-secretase, ApoE, tau metabolism, and immune function are elements within the pathophysiology of AD with identified genetic influences [47]. A coding mutation in the APP gene, for example, results in a 40% reduction in amyloid beta protein (Aβ) formation and a substantial reduction in the risk of AD [48]. This observation supports exploring the use of APP-modifying agents for the treatment and prevention of AD.

Defining the “right target” (or combination of targets) is currently the weakest aspect of AD drug discovery and development. The absence of a deep understanding of AD biology or  focus on inappropriate targets will result in drug development failures regardless of how well the drug development program is conducted. This emphasizes the importance of investment by the National Institutes of Health (NIH), non-US basic biology initiatives, foundations, philanthropists, and others in the fundamental understanding of AD biology and identifying druggable targets and pathways [49].

## The right drug

Clinical drug development is guided by defining a target product profile (TPP) describing the desirable and necessary features of the candidate therapy. The TPP establishes the goals of the development program, and each phase of a program is a step toward fulfilling the TPP [50, 51]. Drugs with TPP-driven development plans have a higher rate of regulatory success than those without [50].

Characterizing a candidate therapy begins with screening assays of the identified target in preclinical discovery campaigns, identifies a lead candidate or limited set of related candidates, continues through establishing the pharmacokinetic (PK) and pharmacodynamic (PD) features in non-clinical animal models, gains refined PK and safety information with first-in-human (FIH) exposure in phase 1 clinical trials, and accrues greater PD and dose-response information in phase 2 trials. Finally, fully powered trials for clinical efficacy are undertaken in phase 3 with efficacy confirmation [52]. Safety data are collected throughout the process.

Preliminary characterization of the molecule as a treatment candidate showing the desired effect in the screening assay starts by determining that it has drug-like properties including molecular weight of ≤ 500 Da, bond features that support membrane penetration including the blood-brain barrier (BBB), no “alerts” that predict toxicity [53, 54], and chemical properties that suggest scalable manufacture and formulation [55, 56]. If the molecule has these encouraging properties, its absorption, distribution, metabolism, excretion, and toxicity (ADMET) are determined in non-clinical models [57].

BBB penetration must be shown in humans in the course of the drug development program during phase 1 [53]. The human BBB has p-glycoprotein transporters and other mechanisms that may not be present in rodents, and central nervous system (CNS) penetration in animal models of AD is not a sufficient guide to human CNS entry [58]. Measurement of CNS levels in non-human primates more closely reflects the human physiology, but direct measures of cerebrospinal fluid (CSF) levels in phase 1 human studies are required in a disciplined drug development program. CSF levels allow the determination of plasma/CSF ratios and help establish whether peripheral levels predict CNS exposures and whether CSF levels are compatible with those showing therapeutic effects in animal models of AD [59, 60]. CSF levels are an acceptable proxy for brain levels but leave some aspects of brain entry, neuronal penetration, and target exposure unassessed [61]. Understanding the PK/PD principles at the site of exposure of the agent to the target is one of the three pillars of drug development proposed by Morgan et al. [17]. Challengesin achievingtarget exposure is one reason for drug development failures in otherwise well-conducted programs. Tarenflurbil, for example, was shown to have poor BBB penetration after the development program was completed [62].

The “right drug” has shown efficacy in non-clinical models of AD. These models have not predicted success in human AD but advancing an agent to human testing without efficacy in animal models would add additional risk to the development program. A common strategy involves using genetic technologies to establish tg species bearing one or more human mutations leading to the overproduction of Aβ [63, 64]. These animals develop amyloid plaques similar to those of human AD but lack neurofibrillary tangles or cell death and are only partial simulacra of human AD [65]. They more closely resemble autosomal dominant AD with mutation-related overproduction of Aβ than typical late-onset AD where clearance of Aβ is the principal underlying problem [66, 67]. Activity in several AD models should be demonstrated to increase confidence in the robustness of the mechanism of the candidate agent [68]. There are recent efforts to more closely model human systems biology using human induced pluripotent stem cell (IPSC) disease models for drug screening [69–71].

Demonstration that the agent has neuroprotective effects is critical to the definition of DMT [33, 52], and interference in the processes leading to cell death should be established prior to human exposure. Many programs have shown effects on Aβ without documenting an impact on neuroprotection; more thorough exploration and demonstration of neuroprotection in non-clinical models may result in agents that exert greater disease modification in human trials.

Phase 1 establishes the PK features and ADMET characteristics of the candidate compound in humans. Several drug doses are assessed, first in single ascending dose (SAD) studies and then in multiple ascending dose (MAD) studies. A maximum tolerated dose (MTD) should be established in phase 1; without this, failure to show efficacy in later stages of development will invariably raise the question of whether the candidate agent was administered at a too-low dose. In some cases, receptor occupancy studies with positron emission tomography (PET), saturation of active transport mechanisms, physical limits on the amount of drug that can be administered, or dose-response curves that remain flat above specific doses obviate the need or the ability to demonstrate an MTD. In all other circumstances, an MTD should be established during phase 1 [72]. MTDs have been difficult to establish for monoclonal antibodies (mAbs), and decisions are often based on feasibility rather than established PK/PD relationships [5]. The decision to increase the doses of mAbs by several folds in recent trials after phase 2 or 3 trials showed no drug-placebo difference (e.g., solanezumab, crenezumab, gantenerumab, aducanumab) demonstrates the difficulty of establishing dose and PK/PD relationships of mAbs; the absence of understanding of PK/PD for mAbs may have contributed to the failure of development programs for these agents. Formulation issues should be resolved prior to evaluating the MTD to ensure that formulation challenges do not prevent the assessment of a full range of doses.

Phase 2 studies establish dose and dose-response relationships. Showing a dose-response association increases confidence in the biological effects of an agent and de-risks further development. The response may be a clinical outcome or a target engagement biomarker linked to the mechanism of action (MOA) of the agent [73–75]. An acceptable dose-response approach includes a low dose with no or little effect, a middle dose with an acceptable biological or clinical outcome, and a high dose that is not well tolerated or raises safety concerns. After the exploration of the dose-response range in phase 2, one or two doses are advanced to phase 3 and will include the final dose(s) of the package insert of information for prescribers and patients. Using a Bayesian dose-finding approach to decide which of 5 BAN2401 doses to advance to phase 3 is an example of dose-finding in phase 2 of a development program [76].

The “right drug” has acceptable toxicity. Safety assessment begins with a review of structural alerts of the molecule predictive of toxicity such as hepatic injury assessed as part of lead candidate nomination and proceeds through evaluations of target organ toxicity in several animal species—typically a rodent species and a dog species [77, 78]. Given an acceptable non-clinical safety profile, the agent is advanced to phase 1 for a FIH assessment of safety in the clinical setting with the determination of the MTD. Safety and tolerability data continue to accrue in phase 2 and phase 3 trials. The number of human exposures remains relatively low until phase 3, and important toxicity observations may be delayed until the late phases of drug development. Semagecestat, avagecestat, and verubecestat were all in phase 3 before cognitive toxicity was identified as an adverse event [79–81]. Some toxicities may not be identified until after approval and widespread human use. Vigilance for toxic effects of agents does not stop with drug approval and continues through the post-approval and marketing period [82]. AD is a fatal illness and—like life-extending cancer therapies—side effects of treatment may be an acceptable trade-off for slowing cognitive decline and maintaining quality of life [83].

The “right drug” at the end of phase 3 has demonstrated the specified features of the TPP, including efficacy and safety, and meets all the requirements for approval by the FDA, the European Medicines Agency (EMA), and other regulatory authorities as an AD therapy [50]. From an industry perspective, the “right” drug has substantial remaining patent life, is competitive with other agents with similar mechanisms, and will be acceptable to payers with reimbursement rates that make the development of the agent commercially attractive [15, 21]. The “right” features of the candidate agent can be scored with a translatability score that allows comparison and prioritization of agents for their readiness to proceed along the translational pathway to human testing and through the phases of clinical trials [84, 85]. Greater use of translational metrics may enhance the likelihood of drug development success [86].

## The right biomarker

Biomarkers play many roles in drug development and are critical to the success of development programs (Table 1) [48]. Including biomarkers in development plans has been associated with greater success rates across therapeutic areas [15, 21, 87]. The use of several types of biomarkers (predictive, prognostic) in development programs is associated with higher success rates in trials compared to trials with no or few biomarkers [88]. The “right” biomarker varies by the type of information needed to inform a development program and the specific phase of drug development. Despite their importance, no biomarker has been qualified by the FDA for use across development programs [89].

**Table 1:** Role of biomarkers in AD drug development

Role in trial	Examples of biomarker used
Identification of trial population	Presence of presenilin 1 (PS1), presenilin 2 (PS2), or amyloid precursor protein (APP) mutations; ApoE-4 plus TOMM40; trisomy 21
Confirmation of diagnosis; exclude non-AD diagnoses	Amyloid imaging; CSF AD signature
Prognosis and course projection	In MCI, ApoE-4 carriers progress more rapidly
Amyloid production and clearance (target engagement)	Stable isotope-labeled kinetics (SILK); BACE activity reduction with BACE inhibitor; CSF Aβ reduction by BACE inhibitor or gamma-secretase inhibitor
Impact of therapy on brain circuit and network function	fMRI; EEG
Impact of therapy on intermediate targets	Amyloid imaging; CSF amyloid; tau PET; CSF phospho-tau
Disease modification	MRI atrophy; CSF total tau; FDG PET; neurofilament light
Stratification for trial analysis	ApoE-4 genotype
Side effect monitoring	MRI surveillance for amyloid-related imaging abnormalities (ARIA); liver function tests; complete blood counts; electrocardiography

The amyloid (A), tau (T), and neurodegeneration (N) framework provides an approach to diagnosis and monitoring of AD and helps guide the choice of biomarkers for drug development [90, 91]. “A” biomarkers (amyloid positron emission tomography [PET], CSF Aβ) support the diagnosis of AD; “A” and “T” (tau PET; CSF phospho-tau) biomarkers are pharmacodynamic biomarkers that can be used to demonstrate target engagement with Aβ or tau species; and “N” (magnetic resonance imaging [MRI], fluorodeoxyglucose PET, CSF total tau) biomarkers are pharmacodynamic markers of neurodegeneration that can provide evidence of neuroprotection and disease modification [33]. Additional markers for “N” are evolving, including neurofilament light (NfL) chain, which has shown promise in multiple sclerosis (MS) trials and preliminary AD trials [92]. Markers of synaptic degeneration such as neurogranin may also contribute to the understanding of therapeutic impact on “N” in AD. Emerging biomarkers are gaining credibility and will add to or amplify the ATN framework applicable to drug development [93].

In AD trials, biomarkers are needed to support the diagnosis. In prevention trials involving cognitively normal individuals, genetic trait biomarkers are used to establish the risk state of the individual or state biomarkers are employed to demonstrate the presence of AD pathology. In trials of treatments for autosomal dominant AD, demonstration of the presenilin 1, presenilin 2, or APP mutation is required in the trial participants [94, 95]. Similarly, in trials involving ApoE-4 homozygotes or heterozygotes or AD in Down syndrome, appropriate testing of chromosome 19 polymorphisms or chromosome 21 triplication is required [96]. A combination of ApoE-4 and TOMM-40 has been used to attempt to show the risk and age of onset of AD [97]. State biomarkers useful in preclinical diagnosis include amyloid PET and the CSF Aβ/tau signature of AD [98, 99]. Tau PET may be useful in identifying individuals appropriate for tau-targeted interventions or for measuring success in reducing the propagation of tau pathology [100].

A substantial number of individuals with a clinical diagnosis of AD have been shown to lack amyloid plaque deposition when studied with amyloid imaging. Forty percent of patients diagnosed clinically with prodromal AD and 25% of those diagnosed with mild AD dementia lack evidence of amyloid pathology when studied with amyloid PET [52, 101]. Those with suspected non-amyloid pathology (SNAP) have undetermined underlying pathology and may not respond to proposed AD therapies. SNAPs may not decline in the expected manner in the placebo group, compromising the ability to demonstrate a drug-placebo difference [102]. SNAPs should be excluded from AD trials; the “right” biomarker for this includes amyloid imaging, the CSF AD signature, or tau imaging in patients with the AD dementia phenotype. In the idalopirdine development program, no enrichment strategies were used and power calculations showed that more than 1600 participants per arm would be needed to show a drug-placebo difference. With enrichment based on amyloid abnormalities, the decline was more rapid and the predicted sample size per arm to show a drug-placebo difference was 148 [103].

Target engagement biomarkers are the “missing link” in many development programs. Having shown that the candidate agent affects the target pathology in preclinical models and is safe in phase 1, sponsors have sometimes advanced through minimal phase 2 studies or directly to phase 3 [22] without showing that the drug treatment has meaningfully engaged the target in humans. Well-conducted phase 2 studies are a critical element of principled drug development and will provide two key pieces of information: target engagement and doses to be assessed in phase 3 [73, 74]. Phase 2 provides the platform for deciding if the candidate agent is viable for further development [75]. Target engagement may be shown directly, for example, with PET receptor occupancy studies or indirectly through proof-of-pharmacology [104, 105]. Examples of proof-of-pharmacology in AD drug development include the demonstration of reduced Aβ production using stable isotope-labeled kinetics (SILK) [106], reduced CSF Aβ with BACE inhibitors [107], glutaminyl cyclase enzyme activity with phosphodiesterase inhibitors [108], and increased Aβ fragments in the plasma and CSF with gamma-secretase inhibitors and modulators [109]. Candidate target engagement/proof-of-pharmacology biomarkers include peripheral indicators of inflammation and oxidation for use in trials of anti-inflammatory and antioxidant compounds. Sponsors of drug development should advance markers of target engagement in concert with the candidate therapy; these may be used after regulatory approval as companion or complementary biomarkers [110, 111]. Demonstration of target engagement does not guarantee efficacy in later stages of development, but target engagement shown by the “right” biomarker provides important de-risking of a candidate treatment by showing biological activity that may translate into clinical efficacy. Semagecestat’s effect on Aβ production in the CSF and aducanumab’s plaque-lowering effect are examples where target engagement was demonstrated in phase 2 or phase 1B, and the agents still failed to show a beneficial drug-placebo difference in later-stage trials [32, 109]. Target engagement and proof-of-pharmacology are “pillars” of successful drug development [17].

Changes in the basic biology of AD—amyloid generation, tau aggregation, inflammation, oxidation, mitochondrial dysfunction, neurodegeneration, etc.—are linked to human cognition through neural circuits whose integrity is critical to normal memory and intellectual function [112]. Two techniques of assessing neural networks are electroencephalography (EEG) and functional magnetic resonance imaging (fMRI). In cognitively normal individuals with positive amyloid PET and low levels of tau as shown by tau PET, fMRI measures of the default mode network (DMN) reveal hyperactive circuit functions. In those with elevated amyloid and elevated tau levels, the circuits become hypoactive compared to age-matched controls [113, 114]. Decline in circuit function predicts progressive cognitive impairment [115]. Disrupted DMN function is present in prodromal AD and in AD dementia [116, 117]. Assessment of DMN integrity may be an important biomarker with predictive value for the impact of the intervention on clinical outcomes [112]. EEG is dependent on the intact network function and may have applications in AD drug development similar to, but more robustly, than those of fMRI [108, 118, 119]. Both EEG and fMRI require procedural and interpretative standardization to be implemented in multi-site trials. A recent alternative for the assessment of circuit integrity in AD is SV2A PET, targeting and visualizing the synaptic network and currently under study as a possible measure of target engagement for drugs aiming to influence synaptic function [120].

Amyloid imaging is a target engagement biomarker establishing reduction of plaque amyloid [111]. Several monoclonal antibodies have shown a dose and time-dependent plaque reduction. In a phase 1B trial, aducanumab achieved both significant plaque reduction and benefit on some clinical measures with evidence of a dose-response relationship [32]. The beneficial effect was not recapitulated in a phase 3 trial. Bapineuzumab and gantenerumab decreased plaque Aβ but had no corresponding impact on cognition or function in the doses studied [121, 122]. Removal of plaque amyloid may be necessary but not sufficient for a therapeutic benefit of anti-amyloid agents or may be a coincidental marker of engagement of a broad range of amyloid species including those required for a therapeutic response. Tau PET assesses target engagement by anti-tau therapeutics; reduced tau burden or reduced tau spread would indicate a therapeutic response [123]. Aβ and tau signals do not measure neuroprotection and are not necessarily evidence of disease modification (DM).

Biomarkers play a critical role in demonstrating DM in DMT development programs. Evidence of neuroprotection is essential to support DM, and structural magnetic resonance imaging (MRI) is the current biomarker of choice for this purpose. Hippocampal atrophy has been linked to progressive disease and to nerve cell loss [124–126]. In clinical trials, MRI has often not fulfilled expectations, and atrophy has sometimes been greater in the treatment groups than in the placebo controls [127, 128]. Recent studies have shown drug-placebo differences on MRI in the anticipated direction suggesting that MRI may be an important DM marker depending on the underlying MOA of the agent. As noted, serum and CSF biomarkers of neurodegeneration such as NfL and synaptic markers have promise to assess successful DMTs but have been incorporated into relatively few AD trials [129]. CSF measures of total tau may be closely related to neurodegeneration and provide useful evidence of the impact on cell death [130, 131].

Biomarkers could eventually have a role as surrogate outcomes for AD trials if they are shown to be predictive of clinical outcomes. Currently, no AD biomarker has achieved surrogate status, and biomarkers are used in concert with clinical outcomes as measures of treatment effects.

Biomarkers have a role in monitoring side effects in the course of clinical trials. Liver, hematologic, and cardiac effects are monitored with liver function tests, complete blood counts, and electrocardiography, respectively. Atabecestat, for example, is a BACE inhibitor whose development was interrupted by the emergence of liver toxicity [132]. Amyloid-related imaging abnormalities (ARIA) of the effusion (ARIA-E) or hemorrhagic (ARIA-H) type may occur with MAbs and are monitored in trials with serial MRI [133]. ARIA has been observed with bapineuzumab, gantenerumab, aducanumab, and BAN2401 [32, 134, 135].

## The right participant

**Fig. 2:**
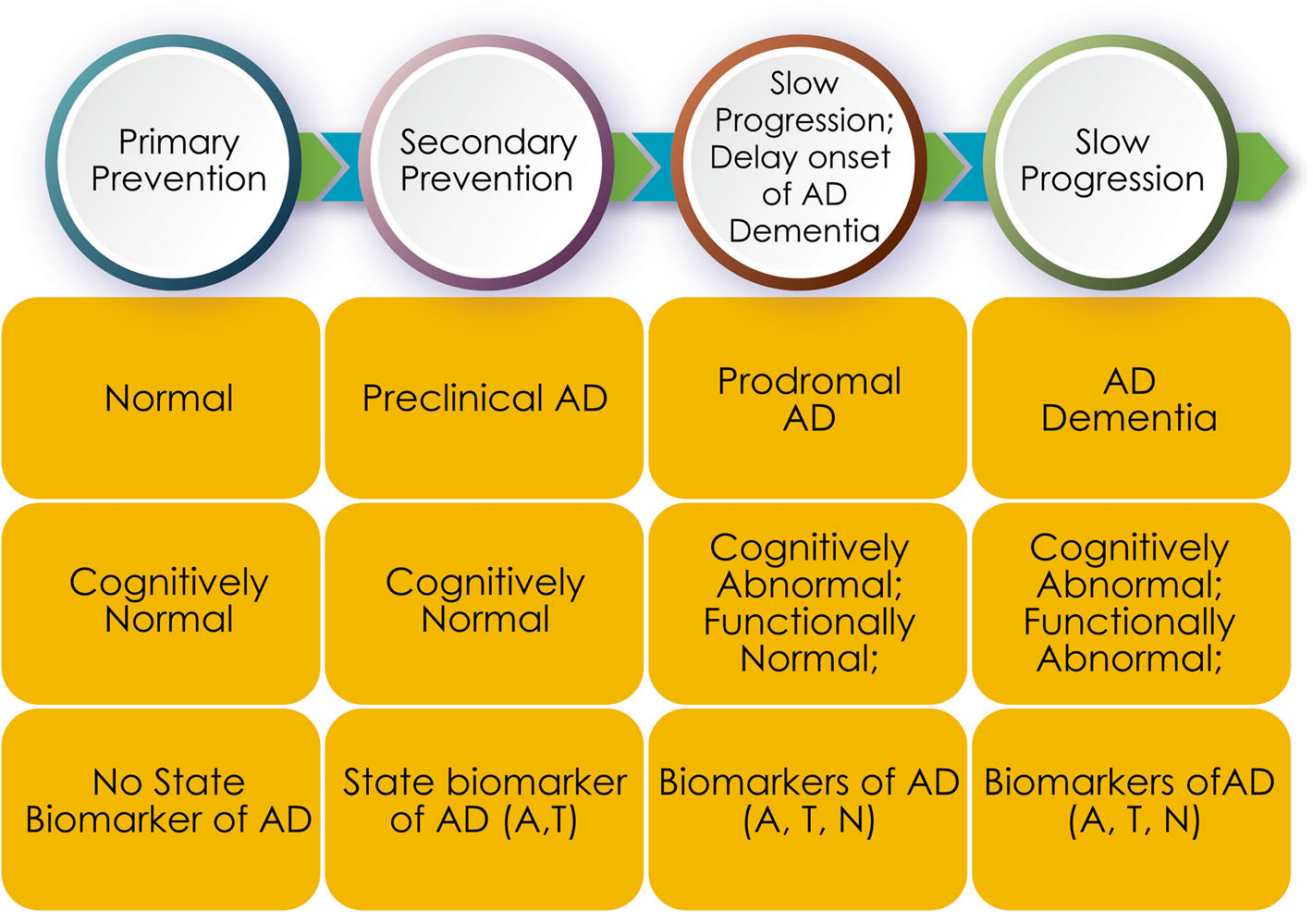
Spectrum of AD and the corresponding cognitive and biomarker state of trial participants (A, amyloid abnormalities; T, tau abnormalities; N, neurodegeneration)

AD progresses through a spectrum of severity from cognitively normal amyloid-bearing preclinical individuals, to those with prodromal AD or prodromal/mild AD dementia and, finally, to those with more severe AD dementia [136, 137] (Fig. 2). Based on this model, trials can target primary prevention in cognitively normal individual with risk factors for AD but no state biomarkers indicative of AD pathology, secondary prevention in preclinical AD participants who are cognitively normal but have positive state biomarkers (positive amyloid PET, low CSF Aβ), and treatment trials aimed at slowing disease progression in prodromal or prodromal/mild AD dementia or mild, moderate, and severe AD dementia (Fig. 2). Although AD represents a seamless progression from unaffected to severely compromised individuals, participants can be assigned to the progressive phases based on genetic markers, cognitive and functional assessments, amyloid imaging or CSF Aβ and tau measures, tau imaging, and MRI [52, 136, 137]. The ATN Framework is designed to guide the identification of the “right” participant for clinical trials [90, 91]. Early intervention has proven to be associated with better outcomes in other disorders such as heart failure [138] suggesting that early intervention in the “brain failure” of AD may have superior outcomes compared to later-phase interventions. However, available cognitive-enhancing agents have been approved for mild, moderate, and severe AD and have failed in trials with predementia participants; some DMT mechanisms may require use earlier in the disease process before pathologic changes are extensive [139–141].

The right participant also relates to the MOA of the agent being assessed. Cognitive enhancing agents will be examined in patients with cognitive abnormalities; agents reducing amyloid production may have the optimal chance of success in primary or secondary prevention; tau prevention trials may focus on the preclinical participants; tau removal agents might be appropriate for prodromal AD or AD dementia; combinations of agents may be assessed in trials with participants with corresponding biomarker changes. Experience with a greater array of agents in a variety of disease phases will help inform the match between the “right” participant and specific agent MOAs. Development of more biomarkers such as those indicating CNS inflammation, excessive oxidation, or the presence of concurrent pathologies such as TDP-43 or alpha-synuclein may assist in matching treatment MOA to the pathological form of AD.

## The right trial

The “right trial” is a well-conducted clinical experiment that answers the central question regarding the superiority of the drug over placebo at the specified dose in the time frame of observation in the defined population. Poorly conducted or underpowered trials do not resolve the central issue of drug efficacy and should not be conducted since they involve participant exposures and potential toxicity without the ability to provide valid informative scientific data. Trial sponsors incur the responsibility to report the results of trials to allow the field to progress by learning from the outcome of each experiment. Participants have accepted the risks of unknown drug effects and placebo exposure, and honoring this commitment requires that the learnings from the trial be made available publically [142].

A key element includes a sample size based on thoroughly vetted anticipated effect sizes. Trial simulations are available to model the results of varying effect sizes and the corresponding required population size [143].

Participation criteria critical to the trial success include defining an appropriate population of preclinical, prodromal, or AD dementia using biomarkers as noted above [136, 137]. Other key participation criteria include the absence of non-AD neurologic diagnoses, physical illness incompatible with trial requirements, or use of medications that may interact with the test agents. Fewer exclusions from trials lead to more generalizable results. Inclusion of diverse populations representative of the populations to which the agent will be marketed enhances the generalizability of trial results.

Clinical outcomes will be chosen based on the specific population included in the trial. The Preclinical Alzheimer Cognitive Composite (PACC) and the Alzheimer Preclinical Cognitive Composite (APCC) used in the Alzheimer’s Prevention Initiative, for example, are used as outcomes in studies of preclinical AD [137, 144, 145]. The Clinical Dementia Rating-Sum of Boxes (CDR-sb) is commonly used as an outcome in prodromal AD [146]. The AD Assessment Scale-Cognitive subscale (ADAS-cog) [147] or the neuropsychological test battery (NTB) [148] and the CDR-sb or Clinical Global Impression of Change with Caregiver Input (CIBIC+) are common dual outcomes in trials of mild-moderate AD dementia [40, 146]. The AD Composite Score (ADCOMS) is an analytic approach including items from the CDR-sb, ADAS-cog, and Mini-Mental State Examination (MMSE) that is sensitive to change and drug effects in prodromal AD and mild AD dementia [149]. The severe impairment battery (SIB) is the outcome assessment most commonly used in severe AD [150]. Having tools with sufficient sensitivity to detect drug-placebo differences in predementia phases of AD is challenging. Commonly used tools such as the ADAS-cog were developed for later stages of the disease. Newer instruments such as the PACC and APCC detect changes over time in natural history studies, but their performance in trials is unknown.

The Alzheimer’s Disease Cooperative Study (ADCS) Activities of Daily Living (ADL) scale is commonly used to assess daily function in patients with MCI and mild to severe AD dementia [151]. The Amsterdam Instrumental Activities of Daily Living (IADL) Questionnaire is increasingly employed for this purpose in MCI/prodromal AD and mild AD dementia [152, 153]. Table 2 summarizes the instruments currently used in trials of each major phase of AD.

**Table 2:** Instruments appropriate as the outcome assessments in different phases of AD

Domain	Prevention trials	Prodromal AD trials	AD dementia trials
Cognition	PACC; APCC	NTB	ADAS-cog in mild to moderate AD; SIB in moderate to severe AD
Global/composite	None	CDR-sb; ADCOMS; iADRS	CIBIC+ in shorter trials; CDR-sb in longer trials
Function	None	ADCS ADL MCI scale; Amsterdan IADL scale	ADCS ADL scale
Behavior	NPI	NPI	NPI

*ADAS-cog* Alzheimer’s Disease Assessment Scale-cognitive subscale, *ADCOMS* Alzheimer’s Disease Composite Scale, Alzheimer’s Disease Cooperative Study Activities of Daily Living scale, *APCC* Alzheimer’s Prevention Initiative (API) Composite Cognitive, *CDR-sb* Clinical Dementia Rating-Sum of Boxes, *CIBIC+* Clinical Interview-Based Impression of Change with Caregiver Input, *IADL* Instrumental Activities of Daily Living, *iADRS* Integrated Alzheimer’s Disease Rating Scale, *NPI* Neuropsychiatric Inventory, *NTB* neuropsychological test battery, *PACC* Preclinical Alzheimer Cognitive Composite, *SIB* severe impairment battery

The trial duration may vary from 12 months to 8 years for DMTs or 3–6 months for symptomatic agents based on the anticipated duration of exposure needed to demonstrate a drug-placebo difference. Preclinical trials may involve observing patients for up to 5 years to allow sufficient decline in the placebo group to be able to demonstrate a drug-placebo difference. These trial duration choices are arbitrary; a basic biological understanding linking the changes in the pathology to the duration of drug exposure is lacking. Using an adaptive design approach, it is possible to adjust trial durations based on emerging patterns of efficacy [76, 154]. Adaptive designs may be used to optimize sample size, trial duration, and dose selection and have been successful in trials of chemotherapy and in trials for treatments of diabetes [155]. Adaptive designs are currently in use in the European Prevention of AD (E-PAD), the Dominantly Inherited Alzheimer Network-Treatment Unit (DIAN-TU), and a study of oxytocin in frontotemporal dementia [156]; broad exploration of the approach is warranted [157, 158].

Globalization of clinical trials with the inclusion of trial sites in many countries is a common response to slow recruitment of trial participants. By increasing the number of trial sites, recruitment can be accelerated and drug efficacy demonstrated more promptly. Globalization, however, increases the number of languages and cultures of participants in the trials as well as increasing the heterogeneity of background experience among the trial sites and investigators. These factors may increase measurement variability and make it more difficult to demonstrate a drug-placebo difference [159–161]. The “right trial” will limit these factors by minimizing the number of regions, languages, and trial sites involved. Within diverse countries such as the USA, the inclusion of minority participants is key to insuring the generalizability of the findings from trials [162].

The right trial will include the right doses selected in phase 2 and the right biomarkers as noted above. The biomarker will be chosen to match the questions to be answered for each trial phase. Target engagement biomarkers are critical in phase 2, and DM biomarkers are critical in phase 3 of DMT trials.

The right trial is also efficiently conducted with rapid start-up, certified raters, a central institutional review board (IRB), and timely recruitment of appropriate subjects. Programs such as the Trial-Ready Cohort for Prodromal and Preclinical AD (TRC-PAD), Global Alzheimer Platform (GAP), and the EPAD initiative aim to enhance the efficiency with which trials are conducted [157, 163]. Development of online registries and trial-ready cohorts may accelerate trial recruitment and treatment evaluation [164–166]. Registries have been helpful in trial recruitment to non-AD disorders [167].

Inclusion of the right number of the right participants is of key importance in successfully advancing AD therapeutics. Compared to other fields, there is a reluctance by patients and physicians to participate in clinical trials for a disease that is considered by some to be a part of normal aging. Advocacy groups throughout the world strive to overcome this attitude; success in engaging participants in trials will become more pressing as more preclinical trials involving cognitively normal individuals are initiated. Sample size is related to the magnitude of the detectable effect which is in turn related to the effect size of the agent and the sensitivity of the measurement tool (clinical instruments or biomarkers); these factors require optimization to allow the conduct of trials with feasible sample sizes.

Hallmarks of poorly designed or conducted trials include failure of the placebo group to decline in the course of a trial (assuming an adequate observation period), failure to show separation of the placebo group from an active treatment arm such as donepezil, excessive measurement variability, or low levels of biological indicators of AD such as the percent of ApoE-4 carriers or the presence of fibrillar amyloid on amyloid imaging [22]. Trials with these features would not be expected to detect drug-placebo differences or to inform the drug development agenda.

A well-designed phase 3 trial builds on observations made in phase 2. Drugs have often been advanced to phase 3 based on the interpretation of apparent effects observed in phase 2 unprespecified subgroup analyses that are derived from small non-randomized samples and are rarely if ever reproduced in phase 3 [22].

## Summary and conclusions

AD drug development has had a high rate of failure [7]. In many cases, BBB penetration, dose, target engagement, or rigorous interrogation of early-stage data has not been adequately pursued. Agents have been advanced to phase 3 with little or no evidence of efficacy in phase 2. Better designed and conducted phase 2 studies will inform further development and enable stopping earlier and preserving resources that can be assigned to testing more drugs in earlier stages (preclinical and FIH), as well as promoting better drugs with a greater chance of success to phase 3 [168]. Deep insight into the biology of AD is currently lacking, and predicting drug success will continue to be challenging; optimizing drug development and clinical trial conduct will reduce this inevitable risk of AD treatment development. Table 3 provides a summary of the integration of the “rights” of AD drug development across the phases of the development cycle.

**Table 3:** Five “rights” implemented across the spectrum of drug development

Right element	Target identification	Drug candidate optimization	Non-clinical assessment	Phase 1	Phase 2	Phase 3
Target	Druggable target identified in AD biology		PD effect supported	PD effect may be assessed with biomarkers	PD effect supported by biomarkers	PD effect supported by biomarkers and clinical outcomes
Drug		Chemical properties	ADME; toxicity; efficacy in animals	PK, ADME in healthy volunteers; MTD established; BBB penetration established	PK, PD in AD	PD in AD
Biomarker			Development of biomarkers useful in trials	Toxicity biomarkers	Patient selection; target engagement biomarkers	Patient selection; DM; toxicity; predictive biomarkers
Patient				Healthy volunteers; AD for immuuno-therapy trials	Prodromal AD, AD dementia	High-risk normal subjects; prodromal AD; AD dementia
Trial				Single ascending dose; multiple ascending dose	Drug-placebo difference at endpoint; adaptive designs	Drug-placebo difference at endpoint; adaptive designs; delay to milestone

*AD* Alzheimer’s disease; *ADME* absorption, distribution, metabolism, excretion; *DM* disease modification; *PK* pharmacokinetics; *PD* pharmacodynamic

This “rights” approach to drug development will enable the precision medicine objective of the right drug, at the right dose, for the right patient, at the right time, tested in the right trial [11–13, 16]. Approaches such as these when used in other therapeutic areas have improved the rate of success of drug development in other settings [15, 21]. Adhering to the “rights of AD drug development” will de-risk many of the challenges of drug development and increase the likelihood of successful trials of critically needed new treatments for AD.

## Data Availability

Not applicable.

## Abbreviations

A: Amyloid
Aβ: Amyloid beta protein
AD: Alzheimer’s disease
ADAS-cog: AD Assessment Scale-Cognitive subscale
ADCOMS: AD Composite Score
ADCS: Alzheimer’s Disease Cooperative Study
ADL: Activities of daily living
ADMET: Absorption, distribution, metabolism, excretion, and toxicity
APOE: Apolipoprotein E
APP: Amyloid precursor protein
ARIA: Amyloid-related imaging abnormalities
ARIA-E: Amyloid-related imaging abnormalities effusion
ARIA-H: Amyloid-related imaging abnormalities hemorrhagic
BACE: Beta-site amyloid precursor protein cleavage enzyme
BBB: Blood-brain barrier
CDR-sb: Clinical Dementia Rating-Sum of Boxes
CIBIC+: Clinical Global Impression of Change with Caregiver Input
CNS: Central nervous system
CSF: Cerebrospinal fluid
DIAN-TU: Dominantly Inherited Alzheimer Network-Treatment Unit
DM: Disease modification
DMN: Default mode network
DMT: Disease-modifying therapy
EEG: Electroencephalography
EMA: European Medicines Agency
E-PAD: European Prevention of Alzheimer’s Disease
fMRI: Functional magnetic resonance imaging
FDA: US Food and Drug Administration
FIH: First-in-human
GAP: Global Alzheimer Platform
IADL: Instrumental Activities of Daily Living Questionnaire
IPSC: Induced pluripotent stem cell
IRB: Institutional review board
MAbs: Monoclonal antibodies
MAD: Multiple ascending dose
MCI: Mild cognitive impairment
MMSE: Mini-Mental State Examination
MOA: Mechanism of action
MTD: Maximum tolerated dose
MRI: Magnetic resonance imaging
MS: Multiple sclerosis
N: Neurodegeneration
NfL: Neurofilament light
NIH: National Institutes of Health
NMDA: *N*-methyl-d-asparate
NPI: Neuropsychiatric Inventory
NTB: Neuropsychological test battery
PACC: Preclinical Alzheimer Cognitive Composite
pAD: Prodromal Alzheimer’s disease
PD: Pharmacodynamic
PET: Positron emission tomography
PK: Pharmacokinetic
SAD: Single ascending dose
SIB: Severe impairment battery
SILK: Stable isotope-labeled kinetics
SNAP: Suspected non-amyloid pathology
T: Tau
tg: Transgenic
TOMM-40: Translocase of outer mitochondrial membrane 40
TPP: Target product profile
TRC-PAD: Trial-Ready Cohort for Prodromal and Preclinical AD
USD: US dollars
